# HIV-1 capture and antigen presentation by dendritic cells: enhanced viral capture does not correlate with better T-Cell activation

**DOI:** 10.1186/1742-4690-9-S2-P2

**Published:** 2012-09-13

**Authors:** M Rodriguez-Plata, A Urrutia, S Cardinaud, M Buzon, N Izquierdo-Useros, JG Prado, M Puertas, I Erkizia, P Coulon, S Cedeño, B Clotet, A Moris, J Martinez-Picado

**Affiliations:** 1 AIDS Research Institute IrsiCaixa, Badalona, Spain; 2 INSERM, UMRS-945, Infection and Immunity, Université Pierre et Marie C, Paris, France; 3 INSERM, UMRS-945, Infection and Immunity, Univ. Pierre et Marie Curie, Paris, France

## Background

During HIV-1 infection, dendritic cells (DC) facilitate dissemination of HIV-1 while trying to trigger adaptive antiviral immune responses. We examined whether increased HIV-1 capture in DC matured with lipopolysaccharide (LPS) results in more efficient antigen presentation to HIV-1–specific CD4^+^ and CD8^+^ T cells. In order to block the DC-mediated trans-infection of HIV-1 and maximize antigen loading, we also evaluated a non-infectious integrase-deficient HIV-1 isolate, the HIV_NL4-3ΔIN_.

## Methods

Immature DC (iDC), mature DC (mDC) activated with IL-1β, TNF-α, IL-6, and PGE2 (ITIP) or LPS during viral uptake, and fully mDC matured with ITIP or with LPS for 48 h before viral loading were tested. Antigen presentation to HIV-1-specific CD4^+^ and CD8^+^ T cell clones was quantified by IFN-γ ELISPOT. DC-associated p24^Gag^ HIV-1 and DC-mediated HIV-1 trans-infection were also evaluated in parallel.

## Results

We showed that higher viral capture of DC did not guarantee better antigen presentation or T-cell activation. Greater HIV_NL4-3_ uptake by fully LPS-matured DC resulted in higher viral transmission to target cells but poorer stimulation of HIV-1–specific CD4^+^ and CD8^+^ T cells. Conversely, maturation of DC with LPS during—but not before—viral loading enhanced both HLA-I and HLA-II HIV-1–derived antigen presentation. On the other hand, DC maturation with ITIP during viral uptake only stimulated HIV-1–specific CD8^+^ T cells. Integrase-deficient HIV_NL4-3ΔIN_ was also efficiently captured and presented by DC through HLA-I and HLA-II pathways, but in absence of viral dissemination.

## Conclusion

Hence, DC maturation state, activation stimulus, and time lag between DC maturation and antigen loading impact HIV-1 capture and virus antigen presentation. Our results demonstrate a dissociation between the capacity to capture HIV-1 and to present viral antigens. HIV_NL4-3ΔIN_ seems to be an attractive candidate to be explored. These results provide new insights into DC biology and have implications in the optimization of DC-based immunotherapy against HIV-1 infection.

